# Association of the lipidome with type 1 diabetes and the mediated effect of metabolites: A Mendelian randomization study

**DOI:** 10.1097/MD.0000000000042755

**Published:** 2025-06-13

**Authors:** Yuman Yin, Min Liao, Yunfeng Yu, Gang Hu, Xinyu Yang, Cong Chen, Rong Yu, Yongjun Wu

**Affiliations:** a School of Traditional Chinese Medicine, Hunan University of Chinese Medicine, Changsha, Hunan, China; b People’s Hospital of Ningxiang City, Changsha, Hunan, China; c School of Basic Medicine, Guizhou University of Traditional Chinese Medicine, Guiyang, Guizhou, China; d School of Pharmacy, Hunan University of Chinese Medicine, Changsha, Hunan, China.

**Keywords:** lipidome, mediated analysis, Mendelian randomization, metabolites, type 1 diabetes

## Abstract

The effects of lipidome and their metabolites on type 1 diabetes (T1D) have not been entirely elucidated, and this study aimed to explore the causal effects of lipidome on T1D and the mediated effects of metabolites by Mendelian randomization (MR). Data on lipidome, metabolites, and T1D were obtained from the genome-wide association study, and single nucleotide polymorphisms were screened according to the basic assumptions of MR. Subsequently, inverse variance weighted was used to analyze the causal relationship between lipidome and T1D, as well as the mediated effect of metabolites. Finally, the horizontal pleiotropy, heterogeneity, and robustness of the results were assessed by MR-Egger intercept, Cochran Q, and leave-one-out sensitivity analysis, respectively. The MR analysis revealed that phosphatidylcholine (PC) (O-16:0_20:4) reduced the genetic susceptibility to T1D by increasing myristoyl dihydrosphingomyelin levels (d18:0/14:0) (mediated proportion: 39.10%, mediated effect: −0.021, 95% confidence interval [CI] ‐0.037 to ‐0.005, *P = *.011) and docosahexaenoylcholine levels (mediated proportion: 31.80%, mediated effect: −0.017, 95% CI −0.032 to -0.002, *P* = .027). Additionally, PC (16:1_20:4) reduced the genetic susceptibility to T1D by increasing myristoyl dihydrosphingomyelin (d18:0/14:0) levels (mediated proportion: 64.30%, mediated effect: −0.021, 95% CI −0.039 to -0.003, *P* = .024). MR-Egger intercept showed no horizontal pleiotropy in these results (*P* ≥ .05). Cochran Q demonstrated no significant heterogeneity in the MR results (*P* ≥ .05). Sensitivity analysis indicated that all results were robust. Our findings revealed pathways by which PC (16:1_20:4) reduced the risk of T1D by increasing myristoyl dihydrosphingomyelin and docosahexaenoylcholine levels, as well as PC (O-16:0_20:4) reduced the risk of T1D by increasing myristoyl dihydrosphingomyelin (d18:0/14:0) levels.

## 1. Introduction

Type 1 diabetes (T1D) is an autoimmune disease characterized by autoimmune destruction of pancreatic β-cells.^[1]^ According to epidemiological surveys, approximately 8.4 million people worldwide developed T1D in 2021^[2]^ and its incidence continues to grow.^[3]^ The main pathological process of T1D involves autoimmune pancreatic β-cell destruction, leading to endogenous insulin deficiency and hyperglycemia.^[4]^ Persistent hyperglycemia is a major cause of vascular complications in organs such as the heart, kidneys, and retina, ultimately leading to reduced quality of life and even death.^[5,6]^ Despite the widespread use of insulin as first-line therapy for T1D, some patients still face the threat of severe diabetic complications.^[7]^ It has been reported that approximately 35,000 undiagnosed patients worldwide died within 12 months of presenting symptoms related to T1D in 2021.^[2]^ Although current studies support that T1D is related to factors such as genetics, environment, immune response, and intestinal microorganisms,^[8,9]^ its pathogenesis is still unclear. Therefore, it has been a hot research topic in endocrinology and immunology to explore the pathogenesis mechanism of T1D and develop related drugs.

Lipidome is the total collection of lipid molecules within a biological organism, encompassing their specific structures, quantities, and spatial locations.^[10]^ It includes 8 types of lipids such as fatty acyls, glycerolipids, glycerophospholipids, sphingolipids, steroids, prenols, glycolipids or saccharolipids, and polyketides.^[11]^ Previous studies have linked abnormal lipid metabolism to various diseases such as Alzheimer disease and atherosclerosis.^[12,13]^ As studies progressed, investigators discovered a link between lipidome and autoimmune diseases, noting its potential involvement in the pathogenesis of T1D.^[14,15]^ A previous study from Spain used lipidomic and metabolomic techniques to analyze the lipid and metabolites of patients with T1D. It revealed that after patients with T1D achieved optimal blood glucose control, their serum triglycerides 45:0 and triglycerides 47:1 levels were significantly reduced, whereas dihydro O-acylceramide (18:1/18:0/16:0) and diacylglycerophosphoethanolamine levels increased significantly.^[15]^ However, apart from this study, there is little literature on the role of lipidome and their metabolites in T1D. Moreover, due to the potential influence of confounding variables and reverse causation, the current study cannot adequately explain the relationship between them. Therefore, we need to explore an effective and comprehensive approach to further assess the causal effects of lipidome and their metabolites on T1D.

In recent years, research methods including Mendelian randomization (MR) and meta-analysis have received much attention.^[16–20]^ MR is an epidemiological research method based on genetic variation, widely used to assess causal relationships between exposure variables and disease or other outcomes.^[21]^ Since genetic variants are randomly assigned at meiosis, MR is less susceptible to confounding variables and reverse causality.^[22]^ In recent years, MR has provided a unique perspective on the etiology of autoimmune diseases, including T1D, and provided potential directions for drug development.^[23,24]^ In this study, we used MR to analyze the causal effects of the lipidome on T1D and the mediated effects of metabolites, aiming to provide new insights into the pathogenesis of T1D.

## 2. Materials and methods

### 2.1. Study design

**Figure 1.**
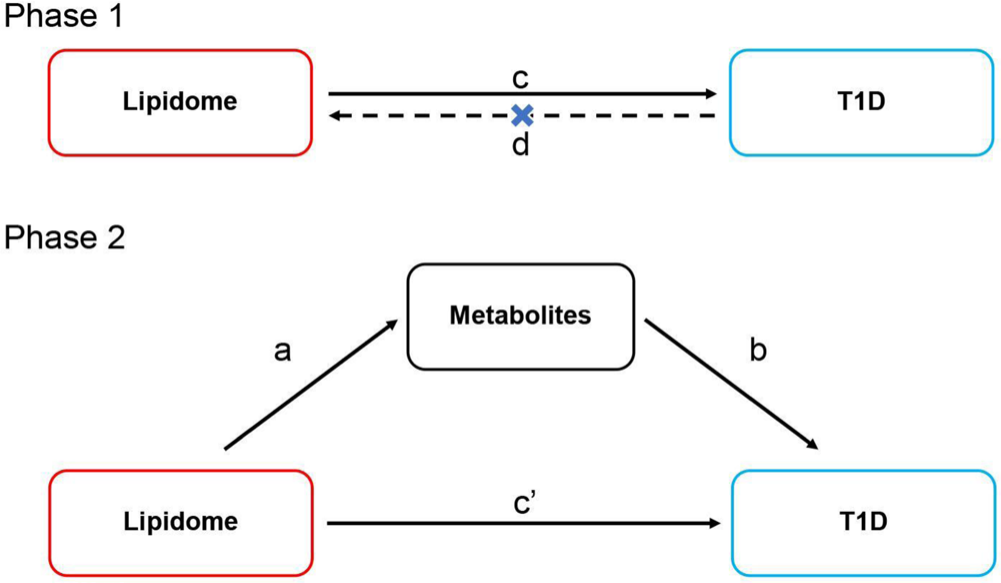
MR design diagrams of lipidome – metabolites – T1D. The a is the effect of lipidome on metabolites; the b is the effect of metabolites on T1D; the c is the total effect of T1D; the c′ is the direct effect of lipidome on T1D; the d is the total effect of T1D on lipidome. The algorithm for the mediated effect is a × b. The algorithm for the mediated proportion is (a × b)/c. MR = Mendelian randomization, T1D = type 1 diabetes.

This MR study is reported according to the Strengthening the Reporting of Observational Studies in Epidemiology Using Mendelian Randomization guidelines.^[25]^ It comprised 2 phases, as shown in Fig. 1. Phase 1: We used bidirectional MR to examine the causal link between the lipidome and T1D. We aimed to find a significant causal impact of the lipidome on T1D. Phase 2: Two-sample MR was employed to evaluate the mediated effect of metabolites in the influence of the lipidome on T1D. This required a significant causal effect of the lipidome on metabolites and a significant causal effect of metabolites on T1D.

### 2.2. Data sources

The data for lipidome were obtained from the GeneRISK cohort of 7174 Finns, including 179 lipid species in 13 lipid classes.^[26]^ The data for metabolites were derived from the Canadian Longitudinal Study on Aging cohort of 8299 individuals of European descent and included 1091 metabolites and 309 metabolite ratios.^[27]^ The data for T1D were sourced from FinnGen (dataset ID: finngen_R10_T1D) (www.finngen.fi/fi), comprising 339,432 Europeans. The definition of T1D in FinnGen is as follows: a chronic condition characterized by minimal or absent production of insulin by the pancreas. Since these data are publicly available, no additional ethical review was required.

### 2.3. Genetic instrumental variables selection

We employed a rigorous methodology for the selection of genetic instrumental variables to ensure the validity and robustness of our causal inferences. Initially, we identified single nucleotide polymorphisms (SNPs) associated with lipidome and metabolites using a threshold of *P* < 5 × 10^‐5^, while simultaneously searching for SNPs related to T1D with a more stringent threshold of *P* < 5 × 10^‐8^. This dual approach allowed us to focus on SNPs that were not only statistically significant but also biologically relevant to our outcomes of interest. To minimize the effects of linkage disequilibrium, we limited our search to independent SNPs by applying a distance threshold of 10,000 kb and an *R*^2^ threshold of <0.001, ensuring that the selected SNPs were not in strong linkage disequilibrium with one another. We further refined our selection by calculating the *F*-statistic for each SNP, using the formula *F* = [*R*^2^/(1‐*R*^2^)]*[(N‐*K*‐1)/*K*], where *K* is the number of paired samples, N is the total number of samples, and *R*^2^ denotes the cumulative explained variance. We restricted our selection to SNPs with an *F*-statistic >10, indicating a strong correlation with the exposure and minimizing the risk of weak instrument bias. Additionally, we adjusted for allelic orientation by excluding palindromic and ambiguous SNPs, which could lead to directional ambiguity after strand inversion. Palindromic SNPs may become directionally ambiguous after strand inversion due to the complementary nature of the forward and reverse strands. This issue is particularly problematic when the effect allele frequency is close to 50%, making the direction determination even more uncertain. Therefore, for palindromic SNPs with effect allele frequencies near this midpoint, we classify them as ambiguous SNPs and exclude them from the analysis to ensure consistency in the allele directions between exposure and outcome data and to maintain the reliability of the analysis. Finally, we utilized the MR-Pleiotropy RESidual Sum and Outlier method to identify and exclude outlier SNPs with *P* < 1, ensuring the accuracy of our causal inference.

### 2.4. Data analysis

First, two-sample MR was used to analyze the bidirectional causality between lipidome and T1D, in order to find the lipidome that had a significant effect on T1D and was not disturbed by reverse causality, thereby obtaining the total effect of lipidome acting on T1D (c). Second, the two-sample MR was conducted to further analyze the causal effect of lipidome on metabolites (a) and metabolites on T1D (b), aiming to find the lipidome and metabolites that satisfy the pathway “lipidome – metabolites – T1D.” The “lipidome metabolites-T1D” pathways refer to the pathways through which lipids regulate T1D genetic susceptibility through metabolites. Third, the mediated effect and mediated proportion of metabolites in lipidome to T1D were calculated based on the above data. The mediated proportion was calculated as (a × b)/c. The above operations were performed by the R 4.3.1 software with the TwoSampleMR (0.5.7) package installed. Inverse variance weighted, a method to achieve unbiased analysis in the lack of pleiotropy, was employed as the primary tool for MR analysis. Additionally, weighted median, an analytical method sensitive to outliers, and MR-Egger, a method for analyzing data in the presence of pleiotropy, were set as secondary tools for MR analysis. MR-Egger was also employed for the assessment of horizontal pleiotropy, which was not present in the results at *P* ≥ .05. Cochran Q and leave-one-out were chosen for heterogeneity analysis and sensitivity analysis of MR results, respectively. The results were not heterogeneous when *P* ≥ .05, and robust when the combined effect sizes were not significantly altered.

## 3. Results

The MR analysis reported 3 pathways of “lipidome – metabolites – T1D.” It contained the causal effects of 3 “lipidome – metabolites,” 2 “metabolites – T1D,” and 2 “lipidome – T1D.” The relevant SNPs are detailed in Tables S1–S7, Supplemental Digital Content, https://links.lww.com/MD/P165.

### 3.1. Lipidome: metabolites

**Figure 2.**
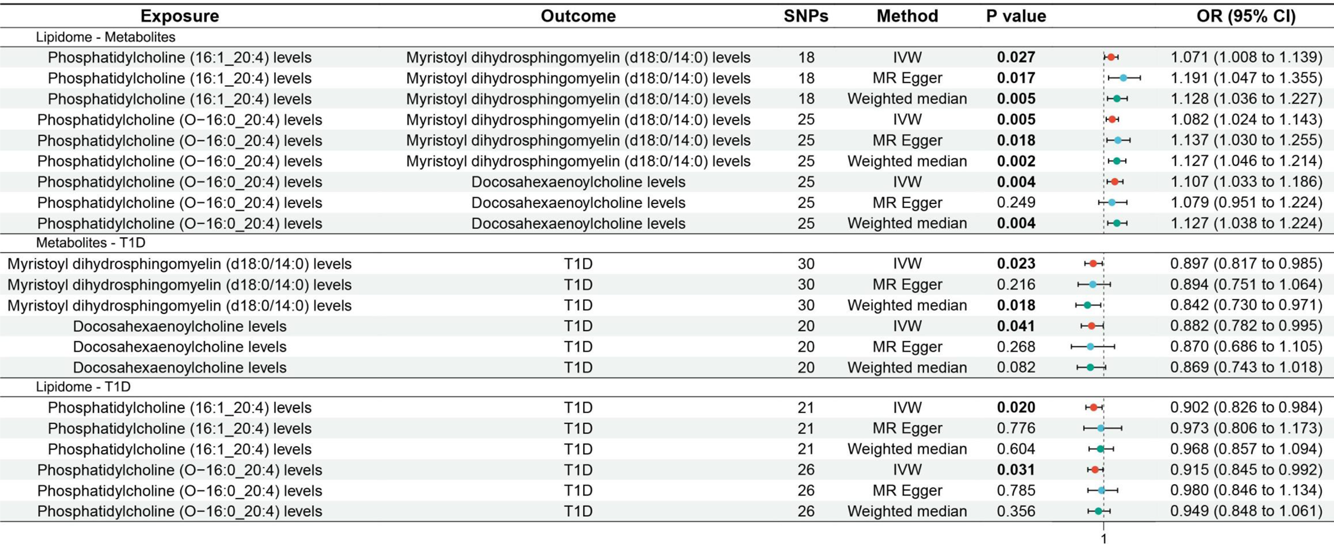
Forest plot of MR analysis for lipidome – metabolites – T1D. MR = Mendelian randomization, T1D = type 1 diabetes.

The MR analysis revealed that phosphatidylcholine (PC) (16:1_20:4) levels (odds ratio [OR] 1.071, 95% confidence interval [CI] 1.008 to 1.139, *P* = .027) and PC (O-16:0_20:4) levels (OR 1.082, 95% CI 1.024 to 1.143, *P* = .005) were associated with increased genetic susceptibility to myristoyl dihydrosphingomyelin (d18:0/14:0) levels. Additionally, PC (O-16:0_20:4) levels were associated with increased genetic susceptibility to docosahexaenoylcholine levels (OR 1.107, 95% CI 1.033 to 1.186, *P* = .004). The forest plot is presented in Fig. 2 and the scatter plot is illustrated in Fig. S1A–C, Supplemental Digital Content, https://links.lww.com/MD/P166. MR-Egger indicated no horizontal pleiotropy of results (*P* ≥ .05), as shown in Table S8, Supplemental Digital Content, https://links.lww.com/MD/P165.

### 3.2. Metabolites: T1D

The MR analysis demonstrated that myristoyl dihydrosphingomyelin (d18:0/14:0) levels (OR 0.897, 95% CI 0.817–0.985, *P* = .023) and docosahexaenoylcholine levels (OR 0.882, 95% CI 0.782–0.995, *P* = .041) were associated with reduced genetic susceptibility to T1D. Forest plot is shown in Fig. 2 and scatter plot is shown in Fig. S1D–E, Supplemental Digital Content, https://links.lww.com/MD/P166. MR-Egger showed no horizontal pleiotropy (*P *≥ .05) of results, as shown in Table S8, Supplemental Digital Content, https://links.lww.com/MD/P165.

### 3.3. Lipidome: T1D

The MR analysis indicated that PC (16:1_20:4) levels (OR 0.902, 95% CI 0.826 to 0.984, *P* = .020) and PC (O-16:0_20:4) levels (OR 0.915, 95% CI 0.845 to 0.992, *P* = .031) were associated with reduced genetic susceptibility to T1D. Forest plot is depicted in Fig. 2 and scatter plot is shown in Fig. S1F–G, Supplemental Digital Content, https://links.lww.com/MD/P166. MR-Egger indicated no horizontal pleiotropy (*P* ≥ .05) of results, as shown in Table S8, Supplemental Digital Content, https://links.lww.com/MD/P165.

### 3.4. Lipidome: metabolites: T1D

**Figure 3.**
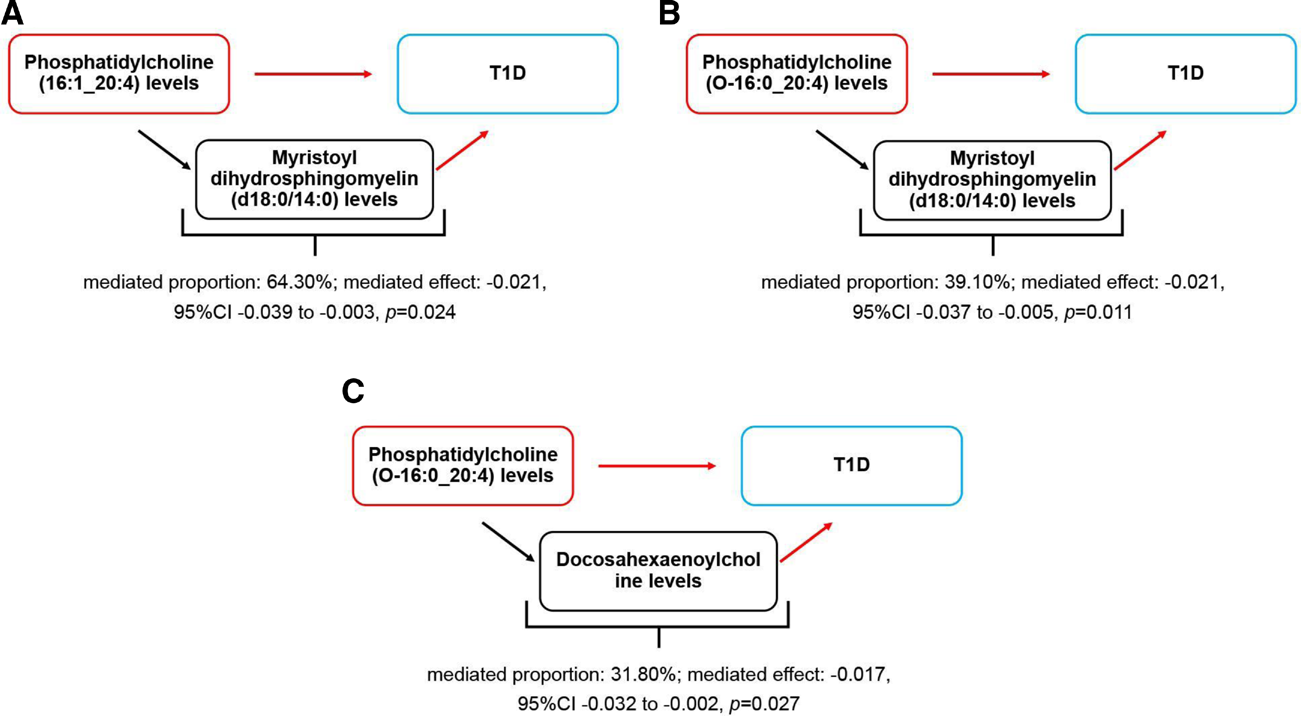
Schematic diagram of the metabolites mediation effect. (A) The mediated effect of myristoyl dihydrosphingomyelin (d18:0/14:0) levels between phosphatidylcholine (16:1_20:4) levels and T1D; (B) the mediated effect of myristoyl dihydrosphingomyelin (d18:0/14:0) levels between phosphatidylcholine (O-16:0_20:4) levels and T1D; (C) the mediated effect of docosahexaenoylcholine levels between phosphatidylcholine (O-16:0_20:4) levels and T1D. The black arrow indicates that the exposure promoted the outcome, while the red arrow indicates that the exposure suppressed the outcome. T1D = type 1 diabetes.

The MR analysis reported 3 pathways of “lipidome – metabolites – T1D,” as depicted in Fig. 3. Among them, PC (16:1_20:4) levels reduced the genetic susceptibility to T1D by increasing myristoyl dihydrosphingomyelin (d18:0/14:0) levels. Myristoyl dihydrosphingomyelin (d18:0/14:0) levels accounted for 64.30% of the reduction in the genetic susceptibility to T1D associated with PC (16:1_20:4) (mediated proportion: 64.30%, mediated effect: −0.021, 95% CI −0.039 to -0.003, *P* = .024).

PC (O-16:0_20:4) levels reduced the genetic susceptibility to T1D by increasing myristoyl dihydrosphingomyelin (d18:0/14:0) levels. Myristoyl dihydrosphingomyelin (d18:0/14:0) levels accounted for 39.10% of the reduction in the genetic susceptibility to T1D associated with PC (O-16:0_20:4) (mediated proportion: 39.10%, mediated effect: −0.021, 95% CI −0.037 to -0.005, *P* = .011).

PC (O-16:0_20:4) levels reduced the genetic susceptibility to T1D by increasing docosahexaenoylcholine levels. Docosahexaenoylcholine levels accounted for 31.80% of the reduction in the genetic susceptibility to T1D associated with PC (O-16:0_20:4) (mediated proportion: 31.80%, mediated effect: −0.017, 95% CI −0.032 to -0.002, *P* = .027).

### 3.5. Heterogeneity and sensitivity analysis

Cochran Q revealed no heterogeneity (*P* ≥ .05) in the MR results, as shown in Fig. S2, Supplemental Digital Content, https://links.lww.com/MD/P166 and Table S9, Supplemental Digital Content, https://links.lww.com/MD/P165. Sensitivity analysis indicated that the MR results were robust, as shown in Fig. S3, Supplemental Digital Content, https://links.lww.com/MD/P166.

## 4. Discussion

### 4.1. Research background and results

T1D is a chronic autoimmune disease, predominantly affecting children and adolescents.^[28,29]^ It eventually leads to a series of serious complications such as cardiovascular lesions, nephropathy, and retinopathy,^[30]^ posing a significant threat to the health of adolescents. As the study progressed, researchers found significant differences in the lipidome and serum metabolite levels between patients with T1D and the healthy population,^[31]^ suggesting the potential involvement of lipidome and their metabolites in the pathogenesis of T1D. However, due to the limited quality and quantity of evidence, the precise roles of various lipidome and metabolites in T1D have not yet been inadequately elucidated. To our knowledge, this is the first MR analysis to explore the causal effects of the lipidome on T1D and the mediated effects of metabolites. Our findings revealed that PC (16:1_20:4) and PC (O-16:0_20:4) reduced the genetic susceptibility to T1D by increasing myristoyl dihydrosphingomyelin (d18:0/14:0) levels. PC (O-16:0_20:4) also reduced the genetic susceptibility to T1D by increasing docosahexaenoylcholine levels. This discovery provides novel genetic insights into the complex relationship between the lipidome and T1D.

### 4.2. Effect of lipidome on T1D

PC (16:1_20:4) refers to PC with palmitoleic acid (16:1) at the Sn-1 position and arachidonic acid (20:4) at the Sn-2 position. PC (O-16:0_20:4) denotes PC with palmitic acid (O-16:0) at the Sn-1 position and arachidonic acid (20:4) at the Sn-2 position. An early lipidomics study revealed that compared to Non-Obese Diabetic mice that did not progress to T1D, the most significantly reduced lipids in Non-Obese Diabetic mice that progressed to T1D were PC and lysophosphatidylcholine.^[32]^ Subsequently, a study of case–controls from Sweden and a prospective study from Finland found that serum PC levels at birth were associated with the risk of T1D.^[33,34]^ A case–control study involving 76 Swedish children with T1D showed that children diagnosed with T1D before the age of 4 years had significantly lower PC levels in umbilical cord blood than the healthy population.^[33]^ A prospective study involving 56 Finnish children with T1D indicated that serum PC levels at birth were lower in children with T1D than in the non-T1D population.^[34]^ Two other studies from Finland also reported significantly lower PC levels in children with T1D compared to non-T1D children with pancreatic antibodies.^[35,36]^ Additionally, a study from Spain further demonstrated that serum PC levels were significantly lower in patients with T1D than in those without T1D, and were not limited to children.^[37]^ These findings support the view that patients with T1D have lower PC levels than the general population, pointing to PC as a protective factor for T1D.

Apart from PC, fatty acids such as arachidonic acid, palmitic acid, and palmitoleic acid have also been reported to be associated with a reduced risk of T1D. First, arachidonic acid serve as an endogenous antidiabetic molecule. A previous animal study demonstrated that plasma arachidonic acid levels in STZ-induced T1D mice were significantly lower than in normal mice.^[38]^ Another clinical study from Spain reported similar results and discovered that arachidonic acid levels in the cell membranes of patients with T1D were significantly lower than those of the general population (12.0% ± 1.6 vs. 15.1% ± 0.6).^[39]^ Subsequent animal experiments demonstrated that arachidonic acid attenuated inflammation and oxidative stress damage in pancreatic islets by lowering tumor necrosis factor-α, interleukin-6, and reactive oxygen species levels, thereby delaying the progression of T1D.^[40,41]^ Second, palmitic acid and palmitoleic acid are associated with a lower risk of T1D. A Finnish study involving 4887 T1D susceptible children found that maternal intake of palmitic acid reduced the risk of T1D in susceptible children by 18% (HR 0.82, 95% CI 0.67–0.99).^[42]^ A subsequent study from Finland indicated that high levels of palmitic acid and palmitoleic acid reduced the autoimmune risk associated with T1D.^[43]^ Another clinical study also revealed that hyperbaric oxygen therapy improved the prognosis of T1D by increasing levels of palmitic acid and palmitoleic acid.^[44]^ These findings suggest that arachidonic acid, palmitic acid, and palmitoleic acid are associated with a reduced risk of T1D, indirectly supporting that PC (16:1_20:4) and PC (O-16:0_20:4) are potential protective factors for T1D.

### 4.3. Effect of metabolites on T1D

Myristoyl dihydrosphingomyelin (d18:0/14:0) denotes a sphingomyelin (SM) formed by the combination of myristoyl (d18:0) and dihydrosphingomyelin (14:0). First, 2 early experimental studies suggested that SM levels were significantly lower in T1D mice than in non-T1D mice.^[32,45]^ Subsequently, a lipidomic study from Spain indicated that SM levels were significantly lower in patients with T1D compared to the healthy population.^[37]^ Similar results were reported in another study from the United States, which found that women with T1D had significantly lower levels of SM in the plasma fraction containing HDL.^[46]^ These findings support that T1D is associated with reduced SM levels, suggesting that SM may be a potential protective factor for T1D. Second, there are some potential relationships between dihydrosphingomyelin and T1D. A clinical study in Spain indicated that serum levels of dihydrosphingomyelins were significantly increased in patients with T1D after optimal glycaemic control was achieved.^[15]^ It revealed that dihydrosphingomyelins were associated with improved prognosis in T1D, suggesting that dihydrosphingomyelins may be a protective factor for T1D. Furthermore, although there is no literature on the association of myristoyl with T1D, myristoylated Akt has been reported to be associated with improved prognosis in T1D mice.^[47]^ Additionally, 1-myristoyl-2-arachidonoyl-GPC was reported to have a role in preserving residual β-cell function.^[48]^ These findings suggest that SM, dihydrosphingomyelin, and myristoyl are associated with a reduced risk of T1D, pointing to myristoyl dihydrosphingomyelin (d18:0/14:0) as a potential protective factor for T1D.

Docosahexaenoylcholine refers to the compound formed by the combination of docosahexaenoic acid (DHA) with choline.^[49]^ A clinical study from the United Kingdom revealed that DHA levels in plasma choline phosphatidylglycerol diglycerides were significantly lower in patients with T1D than in the nondiabetic population (3.7 ± 0.9% vs. 5.2 ± 1.6%).^[50]^ Additionally, cod liver oil, an important source of DHA, has been demonstrated to be associated with T1D.^[51]^ A case–control study from Norway included 545 children with T1D and 1668 random individuals, and found that the intake of cod liver oil during the first year of birth reduced the risk of T1D by 26%.^[51]^ These findings support the association of DHA with a lower risk of T1D, pointing to the possibility that docosahexaenoylcholine may be a protective factor for T1D.

### 4.4. Effect of lipidome-regulated metabolites on T1D

This MR analysis demonstrated that PC (16:1_20:4) and PC (O-16:0_20:4) reduced the genetic susceptibility to T1D by increasing myristoyl dihydrosphingomyelin (d18:0/14:0) levels. Myristoyl dihydrosphingomyelin (d18:0/14:0) accounted for 64.30% and 39.10% of the reduction in the genetic susceptibility to T1D associated with PC (16:1_20:4) and PC (O-16:0_20:4), respectively. Previous studies indicated that PC is involved in the synthesis of myristoyl dihydrosphingomyelin (d18:0/14:0).^[52–54]^ First, the phospholipid acyl group of PC is catalytically transferred to sphingosine by PC transferase, thereby participating in the synthesis of SM.^[52]^ Second, sphingosine synthase 1 and 2 generate diacylglycerol and phosphorylcholine by hydrolysis of PC,^[53]^ which binds to ceramide and ultimately forms SM.^[54]^ Therefore, we speculate that PC (16:1_20:4) and PC (O-16:0_20:4), may promote the synthesis of myristoyl dihydrosphingomyelin (d18:0/14:0) by acting as a substrate for SM, thus reducing the genetic susceptibility to T1D.

This MR analysis also revealed that PC (O-16:0_20:4) reduced the genetic susceptibility to T1D by increasing docosahexaenoylcholine levels. Docosahexaenoylcholine accounts for 31.80% of the reduction in the genetic susceptibility to T1D associated with PC (O-16:0_20:4). Docosahexaenoylcholine, also known as DHA-PC, is formed by combining PC with DHA.^[55]^ Therefore, we speculate that, as a substrate of docosahexaenoylcholine, PC (O-16:0_20:4) may reduce T1D risk by increasing docosahexaenoylcholine levels.

### 4.5. Limitations and prospects

While this study provides novel genetic insights into the “lipidome – metabolites – T1D” pathways, several limitations warrant consideration. First, as the genome-wide association study (GWAS) data were predominantly derived from European populations, our findings may lack generalizability to other ethnic groups. To address this, future studies should prioritize cross-ethnic validation using multi-ancestry cohorts (e.g., African, East Asian, or admixed populations) and employ cross-population MR frameworks to evaluate the trans-ethnic consistency of causal effects. Second, the reliance on summary-level GWAS data inherently precludes access to individual-level covariates (e.g., age, sex, or disease subtypes), which limits our ability to explore nonlinear relationships or perform subgroup analyses. Unfortunately, this constraint is intrinsic to publicly available GWAS datasets, as privacy protections restrict the sharing of granular individual-level data. Future efforts could circumvent this by collaborating with consortia to design prospective studies integrating individual-level omics and clinical data, or by applying nonlinear MR methods to summary statistics. Third, while our MR analysis identified 3 key pathways, these findings require experimental validation to elucidate their biological mechanisms. We propose targeted experiments to confirm pathway causality and identify therapeutic targets. Additionally, translating these findings into clinical practice necessitates bridging the gap between genetic associations and functional biology. For instance, the protective effects of specific PC could be explored as biomarkers for early T1D risk stratification or as dietary supplements in high-risk populations, though rigorous randomized controlled trials are needed to evaluate efficacy. Finally, expanding GWAS to diverse ancestries, integrating single-cell multi-omics data, and fostering interdisciplinary collaborations will be critical to unraveling the spatiotemporal dynamics of lipid-metabolite interactions in T1D pathogenesis.

## 5. Conclusion

This MR analysis demonstrated that PC (16:1_20:4) reduced the genetic susceptibility to T1D by increasing myristoyl dihydrosphingomyelin (d18:0/14:0) levels and docosahexaenoylcholine levels, and PC (O-16:0_20:4) reduced the genetic susceptibility to T1D by increasing myristoyl dihydrosphingomyelin (d18:0/14:0) levels. However, these mechanisms need to be further validated through animal experiments and clinical practice.

## Author contributions

**Conceptualization:** Yunfeng Yu.

**Data curation:** Yuman Yin.

**Methodology:** Yuman Yin, Min Liao, Yongjun Wu.

**Supervision:** Yunfeng Yu, Yongjun Wu.

**Writing – original draft:** Yuman Yin, Yunfeng Yu, Gang Hu, Xinyu Yang.

**Writing – review & editing:** Min Liao, Cong Chen, Rong Yu, Yongjun Wu.

## Supplementary Material


