# Supplementary material for: Association of the lipidome with type 1 diabetes and the mediated effect of metabolites: A Mendelian randomization study
Source: Medicine (Baltimore). 2025 Jun 13;104(24):e42755. doi: 10.1097/MD.0000000000042755 (PMC12173266; doi:10.1097/MD.0000000000042755)
Supplement: Supplementary file 1 [file medi-104-e42755-s001.pdf]

Supplementary Table S1 Information of identified SNPs in Phosphatidylcholine (16:1_20:4) levels and Myristoyl dihydrosphingomyelin (d18:0/14:0) levels

	Exposure (Phosphatidylcholine (16:1_20:4) levels)					Outcome (Myristoyl dihydrosphingomyelin (d18:0/14:0) levels)			
SNP	EA	OA	β	SE	*p* value	Samplesize	β	SE	*p* value
rs10848122	C	G	-0.110	0.023	2.53609E-06	8,254	0.002	0.017	0.905992
rs11650558	T	C	0.092	0.019	1.4908E-06	8,254	-0.005	0.015	0.717927
rs11659363	G	T	0.104	0.022	2.59634E-06	8,254	-0.023	0.018	0.204236
rs117505687	A	G	-0.226	0.051	9.35726E-06	8,254	-0.004	0.036	0.903112
rs174535	C	T	-0.328	0.019	5.3348E-64	8,254	-0.056	0.016	0.000293834
rs1757973	A	G	0.093	0.021	9.53963E-06	8,254	-0.022	0.017	0.193431
rs192436652	T	C	0.203	0.043	2.79598E-06	8,254	-0.030	0.047	0.526946
rs2197750	T	C	0.164	0.037	7.74175E-06	8,254	-0.002	0.044	0.95606
rs2446624	T	C	0.163	0.035	2.79605E-06	8,254	0.003	0.028	0.905296
rs35811605	T	A	0.138	0.029	2.25271E-06	8,254	-0.003	0.022	0.887574
rs56317095	A	G	0.269	0.056	1.62021E-06	8,254	-0.014	0.040	0.725211
rs591931	T	C	0.110	0.024	5.55074E-06	8,254	0.000	0.020	0.998035
rs6545883	G	A	0.095	0.020	1.16604E-06	8,254	0.021	0.015	0.16815
rs695112	T	A	-0.109	0.022	1.33436E-06	8,254	-0.018	0.020	0.359136
rs75781049	G	T	0.169	0.038	9.12825E-06	8,254	0.004	0.026	0.886209
rs75878220	C	G	-0.306	0.062	7.31569E-07	8,254	0.026	0.044	0.55401
rs8068175	T	C	0.089	0.019	3.3161E-06	8,254	0.008	0.016	0.593267
rs8100204	A	G	-0.126	0.026	9.86769E-07	8,254	-0.014	0.022	0.514341

SNP, single nucleotide polymorphism; EA, effect allele; OA, other allele; SE, standard errors.

Supplementary Table S2 Information of identified SNPs in Phosphatidylcholine (O-16:0_20:4) levels and Myristoyl dihydrosphingomyelin (d18:0/14:0) levels

			Exposure (Phosphatidylcholine (O-16:0_20:4) levels)			Outcome (Myristoyl dihydrosphingomyelin (d18:0/14:0) levels)			
SNP	EA	OA	β	SE	*p* value	Samplesize	β	SE	*p* value
rs10867645	T	C	-0.078	0.017	5.21884E-06	8,254	-0.023	0.015	0.140144
rs11057853	C	T	-0.088	0.017	1.68854E-07	8,254	-0.017	0.015	0.261978
rs11173828	A	G	-0.160	0.033	1.16925E-06	8,254	0.002	0.034	0.949676
rs114653320	G	A	0.278	0.062	7.51335E-06	8,254	0.071	0.052	0.169045
rs115076819	T	C	-0.222	0.050	9.01887E-06	8,254	0.023	0.034	0.496323
rs117215929	C	T	-0.200	0.043	3.27117E-06	8,254	0.025	0.050	0.62709
rs12928189	G	A	-0.078	0.018	8.41124E-06	8,254	0.020	0.017	0.247191
rs145755646	G	C	0.184	0.034	7.23372E-08	8,254	-0.002	0.034	0.94451
rs146605342	A	G	0.197	0.044	8.17627E-06	8,254	-0.042	0.066	0.52182
rs173539	T	C	0.089	0.018	1.63435E-06	8,254	0.001	0.015	0.968365
rs174568	T	C	-0.382	0.016	1.1577E-114	8,254	-0.049	0.016	0.00182313
rs2678932	C	T	-0.081	0.017	1.07956E-06	8,254	-0.009	0.015	0.57807
rs2829725	T	A	0.081	0.018	7.18496E-06	8,254	0.007	0.016	0.65025
rs2903910	A	T	0.202	0.039	3.17188E-07	8,254	0.033	0.035	0.340981
rs34881711	A	G	0.107	0.020	6.84329E-08	8,254	-0.032	0.018	0.0767323
rs3741252	T	C	0.139	0.025	1.697E-08	8,254	0.007	0.031	0.818566
rs642327	G	C	-0.077	0.017	9.67706E-06	8,254	-0.008	0.015	0.57927
rs6601694	A	G	0.083	0.018	3.03899E-06	8,254	0.005	0.016	0.745216
rs6709442	A	G	0.095	0.017	3.00419E-08	8,254	0.029	0.015	0.0543288
rs698909	A	G	0.082	0.018	6.00342E-06	8,254	-0.006	0.016	0.698249
rs73422593	G	A	-0.130	0.027	1.68215E-06	8,254	0.007	0.025	0.788584
rs75385811	G	A	-0.095	0.021	5.76552E-06	8,254	0.016	0.018	0.384115
rs7554873	C	T	0.186	0.039	2.12774E-06	8,254	0.030	0.033	0.367515
rs76798309	A	G	0.298	0.065	4.3564E-06	8,254	0.016	0.042	0.70146
rs7982529	C	T	0.092	0.020	4.30169E-06	8,254	-0.003	0.018	0.847243

SNP, single nucleotide polymorphism; EA, effect allele; OA, other allele; SE, standard errors.

Supplementary Table S3 Information of identified SNPs in Phosphatidylcholine (O-16:0_20:4) levels and Docosahexaenoylcholine levels

	Exposure (Phosphatidylcholine (O-16:0_20:4) levels)					Outcome (Docosahexaenoylcholine levels)			
SNP	EA	OA	β	SE	*p* value	Samplesize	β	SE	*p* value
rs10867645	T	C	-0.078	0.017	5.21884E-06	7,440	-0.017	0.017	0.311796
rs11057853	C	T	-0.088	0.017	1.68854E-07	7,440	-0.021	0.016	0.209078
rs11173828	A	G	-0.160	0.033	1.16925E-06	7,440	-0.004	0.037	0.922847
rs114653320	G	A	0.278	0.062	7.51335E-06	7,440	-0.030	0.056	0.591508
rs115076819	T	C	-0.222	0.050	9.01887E-06	7,440	-0.105	0.038	0.00516902
rs117215929	C	T	-0.200	0.043	3.27117E-06	7,440	0.076	0.055	0.170227
rs12928189	G	A	-0.078	0.018	8.41124E-06	7,440	-0.002	0.019	0.910815
rs145755646	G	C	0.184	0.034	7.23372E-08	7,440	-0.094	0.038	0.0126348
rs146605342	A	G	0.197	0.044	8.17627E-06	7,440	-0.102	0.072	0.155833
rs173539	T	C	0.089	0.018	1.63435E-06	7,440	-0.012	0.017	0.465322
rs174568	T	C	-0.382	0.016	1.1577E-114	7,440	-0.047	0.017	0.00544625
rs2678932	C	T	-0.081	0.017	1.07956E-06	7,440	-0.027	0.017	0.11038
rs2829725	T	A	0.081	0.018	7.18496E-06	7,440	0.030	0.018	0.0945131
rs2903910	A	T	0.202	0.039	3.17188E-07	7,440	0.008	0.038	0.837927
rs34881711	A	G	0.107	0.020	6.84329E-08	7,440	0.004	0.020	0.847868
rs3741252	T	C	0.139	0.025	1.697E-08	7,440	0.021	0.034	0.544513
rs642327	G	C	-0.077	0.017	9.67706E-06	7,440	-0.019	0.017	0.263175
rs6601694	A	G	0.083	0.018	3.03899E-06	7,440	0.021	0.018	0.237193
rs6709442	A	G	0.095	0.017	3.00419E-08	7,440	-0.005	0.016	0.766762
rs698909	A	G	0.082	0.018	6.00342E-06	7,440	0.020	0.017	0.245037
rs73422593	G	A	-0.130	0.027	1.68215E-06	7,440	-0.008	0.027	0.754402
rs75385811	G	A	-0.095	0.021	5.76552E-06	7,440	-0.034	0.020	0.0923671
rs7554873	C	T	0.186	0.039	2.12774E-06	7,440	-0.012	0.036	0.746522
rs76798309	A	G	0.298	0.065	4.3564E-06	7,440	0.039	0.046	0.394952
rs7982529	C	T	0.092	0.020	4.30169E-06	7,440	-0.008	0.019	0.665866

SNP, single nucleotide polymorphism; EA, effect allele; OA, other allele; SE, standard errors.

Supplementary Table S4 Information of identified SNPs in Myristoyl dihydrosphingomyelin (d18:0/14:0) levels and T1D

	Exposure (Myristoyl dihydrosphingomyelin (d18:0/14:0) levels)					Outcome (T1D)			
SNP	EA	OA	β	SE	*p* value	Samplesize	β	SE	*p* value
rs10129238	T	C	-0.074	0.017	9.30273E-06	339,432	0.035	0.026	0.171628
rs10423572	C	T	0.174	0.036	1.43027E-06	339,432	0.002	0.123	0.98849
rs11112344	T	C	-0.097	0.022	9.31441E-06	339,432	0.008	0.036	0.819254
rs117621282	C	T	0.212	0.041	2.50665E-07	339,432	-0.051	0.060	0.401251
rs12437952	A	C	0.093	0.021	9.84946E-06	339,432	-0.001	0.028	0.964014
rs1267212	G	T	0.106	0.024	6.18788E-06	339,432	-0.040	0.042	0.351877
rs12988381	T	C	0.085	0.019	7.41929E-06	339,432	-0.004	0.030	0.883226
rs139690031	T	C	-0.298	0.064	3.02073E-06	339,432	0.021	0.056	0.714407
rs149510848	A	G	-0.331	0.069	1.51258E-06	339,432	-0.114	0.081	0.156859
rs150643464	G	A	-0.191	0.040	1.44942E-06	339,432	0.018	0.061	0.763889
rs16839821	C	A	-0.104	0.022	2.71435E-06	339,432	-0.027	0.034	0.424085
rs17512296	T	C	0.079	0.018	7.05524E-06	339,432	-0.008	0.026	0.773115
rs2448907	C	A	-0.188	0.040	2.89561E-06	339,432	-0.024	0.068	0.719459
rs28370010	A	G	0.252	0.056	5.71288E-06	339,432	0.192	0.124	0.120519
rs340079	C	A	0.068	0.015	8.62745E-06	339,432	0.037	0.024	0.117339
rs34847539	C	T	0.078	0.015	3.26476E-07	339,432	-0.030	0.023	0.183985
rs364585	G	A	-0.182	0.015	9.7043E-34	339,432	0.045	0.024	0.0595114
rs4537986	C	T	0.112	0.025	9.22724E-06	339,432	-0.002	0.040	0.956159
rs5755708	C	T	-0.067	0.015	7.31148E-06	339,432	0.029	0.023	0.198467
rs62031328	T	C	0.068	0.015	6.14712E-06	339,432	-0.024	0.024	0.322181
rs62066054	A	G	-0.072	0.016	9.99387E-06	339,432	0.001	0.024	0.976012
rs62094355	T	C	0.111	0.024	4.66924E-06	339,432	0.042	0.032	0.191064
rs6933365	G	A	-0.419	0.089	2.28268E-06	339,432	0.101	0.041	0.0146433
rs71483327	T	C	0.333	0.074	7.26784E-06	339,432	0.024	0.150	0.870035
rs72908201	T	C	-0.125	0.028	7.39821E-06	339,432	0.050	0.045	0.265267
rs74868722	T	C	0.255	0.056	5.08429E-06	339,432	0.036	0.090	0.69026
rs77325406	T	C	0.133	0.028	2.90356E-06	339,432	-0.002	0.035	0.957426
rs78668361	G	A	-0.321	0.068	2.1271E-06	339,432	-0.002	0.080	0.976762
rs9309188	G	C	-0.075	0.017	8.52931E-06	339,432	0.007	0.027	0.795099
rs9519977	A	G	-0.071	0.016	5.92233E-06	339,432	0.012	0.023	0.592748

SNP, single nucleotide polymorphism; EA, effect allele; OA, other allele; SE, standard errors.

Supplementary Table S5 Information of identified SNPs in Docosahexaenoylcholine levels and T1D

Exposure (Docosahexaenoylcholine levels)						Outcome (T1D)			
SNP	EA	OA	β	SE	*p* value	Samplesize	β	SE	*p* value
rs112560306	T	C	-0.426	0.095	7.79515E-06	339,432	0.112	0.102	0.27497
rs113971164	T	C	0.116	0.026	6.06758E-06	339,432	-0.010	0.033	0.766809
rs11880992	A	G	-0.073	0.016	5.89059E-06	339,432	0.026	0.023	0.268358
rs12121447	T	C	-0.111	0.024	3.1417E-06	339,432	-0.045	0.035	0.205055
rs12480856	T	C	-0.169	0.036	3.18212E-06	339,432	0.012	0.042	0.777293
rs13032126	A	T	-0.199	0.045	8.18425E-06	339,432	0.036	0.057	0.531062
rs1437468	C	A	0.223	0.043	2.00747E-07	339,432	-0.069	0.069	0.316626
rs149320805	A	G	0.390	0.086	6.29581E-06	339,432	-0.092	0.134	0.494573
rs17426755	A	T	-0.210	0.043	1.38706E-06	339,432	0.032	0.055	0.554118
rs17608675	T	C	0.074	0.017	8.07569E-06	339,432	-0.013	0.023	0.575655
rs185121193	C	G	0.282	0.062	4.84271E-06	339,432	-0.103	0.064	0.109923
rs1942333	C	T	0.083	0.019	9.48377E-06	339,432	-0.035	0.024	0.146106
rs2054120	A	G	-0.092	0.019	2.09903E-06	339,432	-0.019	0.024	0.437411
rs537178127	A	G	0.380	0.082	3.57328E-06	339,432	0.009	0.075	0.899329
rs72970471	A	G	-0.196	0.044	6.99154E-06	339,432	0.019	0.045	0.673014
rs73688611	T	C	-0.110	0.024	4.53986E-06	339,432	0.031	0.034	0.35203
rs74580783	G	T	-0.285	0.061	2.54538E-06	339,432	-0.054	0.123	0.661806
rs74704440	T	C	-0.252	0.055	4.10032E-06	339,432	0.003	0.072	0.963561
rs869405	T	C	-0.296	0.065	5.40071E-06	339,432	0.059	0.083	0.482268
rs9461383	G	A	-0.077	0.016	2.07705E-06	339,432	0.004	0.022	0.866554

SNP, single nucleotide polymorphism; EA, effect allele; OA, other allele; SE, standard errors.

Supplementary Table S6 Information of identified SNPs in Phosphatidylcholine (16:1_20:4) levels and T1D

Exposure (Phosphatidylcholine (16:1_20:4) levels)						Outcome (T1D)			
SNP	EA	OA	β	SE	*p* value	Samplesize	β	SE	*p* value
rs10848122	C	G	-0.110	0.023	2.53609E-06	339,432	0.014	0.027	0.59696
rs11650558	T	C	0.092	0.019	1.4908E-06	339,432	-0.037	0.023	0.107145
rs11659363	G	T	0.104	0.022	2.59634E-06	339,432	-0.030	0.026	0.250654
rs117505687	A	G	-0.226	0.051	9.35726E-06	339,432	0.099	0.060	0.0994008
rs174535	C	T	-0.328	0.019	5.3348E-64	339,432	0.009	0.023	0.67992
rs1757973	A	G	0.093	0.021	9.53963E-06	339,432	-0.007	0.025	0.773936
rs192436652	T	C	0.203	0.043	2.79598E-06	339,432	-0.030	0.053	0.565969
rs2085061	G	T	0.188	0.041	6.18549E-06	339,432	-0.076	0.048	0.115471
rs2197750	T	C	0.164	0.037	7.74175E-06	339,432	0.033	0.044	0.453169
rs2446624	T	C	0.163	0.035	2.79605E-06	339,432	0.010	0.042	0.8117
rs35811605	T	A	0.138	0.029	2.25271E-06	339,432	-0.053	0.035	0.126893
rs4539990	C	T	-0.103	0.023	9.3081E-06	339,432	0.004	0.027	0.894443
rs56317095	A	G	0.269	0.056	1.62021E-06	339,432	-0.006	0.066	0.930136
rs591931	T	C	0.110	0.024	5.55074E-06	339,432	-0.018	0.029	0.524734
rs6545883	G	A	0.095	0.020	1.16604E-06	339,432	0.023	0.023	0.32688
rs695112	T	A	-0.109	0.022	1.33436E-06	339,432	0.004	0.027	0.887938
rs75781049	G	T	0.169	0.038	9.12825E-06	339,432	-0.104	0.047	0.0268077
rs75878220	C	G	-0.306	0.062	7.31569E-07	339,432	0.021	0.080	0.79337
rs8068175	T	C	0.089	0.019	3.3161E-06	339,432	-0.014	0.023	0.558397
rs8100204	A	G	-0.126	0.026	9.86769E-07	339,432	0.002	0.031	0.941033
rs9809733	A	C	-0.084	0.019	9.73301E-06	339,432	0.025	0.023	0.272328

SNP, single nucleotide polymorphism; EA, effect allele; OA, other allele; SE, standard errors.

Supplementary Table S7 Information of identified SNPs in Phosphatidylcholine (O-16:0_20:4) levels and T1D

	Exposure (Phosphatidylcholine (O-16:0_20:4) levels)					Outcome (T1D)			
SNP	EA	OA	β	SE	*p* value	Samplesize	β	SE	*p* value
rs10867645	T	C	-0.078	0.017	5.21884E-06	339,432	0.034	0.023	0.140534
rs11057853	C	T	-0.088	0.017	1.68854E-07	339,432	-0.006	0.023	0.781779
rs11173828	A	G	-0.160	0.033	1.16925E-06	339,432	0.058	0.046	0.211878
rs114653320	G	A	0.278	0.062	7.51335E-06	339,432	-0.076	0.074	0.30596
rs115076819	T	C	-0.222	0.050	9.01887E-06	339,432	-0.052	0.066	0.432698
rs117215929	C	T	-0.200	0.043	3.27117E-06	339,432	0.053	0.060	0.38338
rs12928189	G	A	-0.078	0.018	8.41124E-06	339,432	-0.023	0.024	0.330595
rs145755646	G	C	0.184	0.034	7.23372E-08	339,432	-0.032	0.047	0.492423
rs146605342	A	G	0.197	0.044	8.17627E-06	339,432	-0.028	0.059	0.629511
rs173539	T	C	0.089	0.018	1.63435E-06	339,432	-0.009	0.025	0.735506
rs174568	T	C	-0.382	0.016	1.1577E-114	339,432	0.014	0.023	0.551346
rs2678932	C	T	-0.081	0.017	1.07956E-06	339,432	0.038	0.023	0.0946237
rs2829725	T	A	0.081	0.018	7.18496E-06	339,432	-0.039	0.025	0.115742
rs2903910	A	T	0.202	0.039	3.17188E-07	339,432	0.061	0.052	0.242251
rs34881711	A	G	0.107	0.020	6.84329E-08	339,432	-0.030	0.027	0.262154
rs3741252	T	C	0.139	0.025	1.697E-08	339,432	-0.086	0.034	0.0117411
rs642327	G	C	-0.077	0.017	9.67706E-06	339,432	0.017	0.023	0.463338
rs658580	A	C	0.090	0.019	3.17741E-06	339,432	-0.007	0.025	0.783091
rs6601694	A	G	0.083	0.018	3.03899E-06	339,432	-0.001	0.024	0.960043
rs6709442	A	G	0.095	0.017	3.00419E-08	339,432	-0.025	0.023	0.280321
rs698909	A	G	0.082	0.018	6.00342E-06	339,432	0.010	0.024	0.685463
rs73422593	G	A	-0.130	0.027	1.68215E-06	339,432	-0.062	0.036	0.0845045
rs75385811	G	A	-0.095	0.021	5.76552E-06	339,432	-0.002	0.029	0.940974
rs7554873	C	T	0.186	0.039	2.12774E-06	339,432	-0.028	0.049	0.566933
rs76798309	A	G	0.298	0.065	4.3564E-06	339,432	-0.059	0.085	0.483246
rs7982529	C	T	0.092	0.020	4.30169E-06	339,432	-0.016	0.027	0.555019

SNP, single nucleotide polymorphism; EA, effect allele; OA, other allele; SE, standard errors.

Supplementary Table S8 MR-Egger intercept analyses of horizontal pleiotropy

Exposure	Outcome	Egger_intercept	SE	*p* value
Phosphatidylcholine (16:1_20:4) levels	Myristoyl dihydrosphingomyelin (d18:0/14:0) levels	-0.01888	0.01037	0.08740419
Phosphatidylcholine (O-16:0_20:4) levels	Myristoyl dihydrosphingomyelin (d18:0/14:0) levels	-0.00858	0.00719	0.244667562
Phosphatidylcholine (O-16:0_20:4) levels	Docosahexaenoylcholine levels	0.00438	0.00918	0.637395554
Myristoyl dihydrosphingomyelin (d18:0/14:0) levels	T1D	0.00056	0.01130	0.961101378
Docosahexaenoylcholine levels	T1D	0.00208	0.01633	0.899802056
Phosphatidylcholine (16:1_20:4) levels	T1D	-0.01282	0.01431	0.38154424
Phosphatidylcholine (O-16:0_20:4) levels	T1D	-0.01149	0.01059	0.288708596

SE, standard errors; T1D, type 1 diabetes.

Supplementary Table S9 Cochran's Q tests of heterogeneity

Exposure	Outcome	Method	Q	Q_df	Q_*p* val
Phosphatidylcholine (16:1_20:4) levels	Myristoyl dihydrosphingomyelin (d18:0/14:0) levels	MR Egger	12.718	16	0.693252347
Phosphatidylcholine (16:1_20:4) levels	Myristoyl dihydrosphingomyelin (d18:0/14:0) levels	Inverse variance weighted	16.033	17	0.521494164
Phosphatidylcholine (O-16:0_20:4) levels	Myristoyl dihydrosphingomyelin (d18:0/14:0) levels	MR Egger	18.879	23	0.708221896
Phosphatidylcholine (O-16:0_20:4) levels	Myristoyl dihydrosphingomyelin (d18:0/14:0) levels	Inverse variance weighted	20.305	24	0.679330963
Phosphatidylcholine (O-16:0_20:4) levels	Docosahexaenoylcholine levels	MR Egger	31.531	23	0.110307903
Phosphatidylcholine (O-16:0_20:4) levels	Docosahexaenoylcholine levels	Inverse variance weighted	31.843	24	0.130926218
Myristoyl dihydrosphingomyelin (d18:0/14:0) levels	T1D	MR Egger	23.714	28	0.696528538
Myristoyl dihydrosphingomyelin (d18:0/14:0) levels	T1D	Inverse variance weighted	23.717	29	0.742777987
Docosahexaenoylcholine levels	T1D	MR Egger	9.586	18	0.944595528
Docosahexaenoylcholine levels	T1D	Inverse variance weighted	9.602	19	0.962091655
Phosphatidylcholine (16:1_20:4) levels	T1D	MR Egger	14.631	19	0.745719304
Phosphatidylcholine (16:1_20:4) levels	T1D	Inverse variance weighted	15.434	20	0.751062368
Phosphatidylcholine (O-16:0_20:4) levels	T1D	MR Egger	22.856	24	0.528301503
Phosphatidylcholine (O-16:0_20:4) levels	T1D	Inverse variance weighted	24.033	25	0.517446722

T1D, type 1 diabetes.
