# Supplementary figures and images for: Association of the lipidome with type 1 diabetes and the mediated effect of metabolites: A Mendelian randomization study
Source: Medicine (Baltimore). 2025 Jun 13;104(24):e42755. doi: 10.1097/MD.0000000000042755 (PMC12173266; doi:10.1097/MD.0000000000042755)

## Slide 1
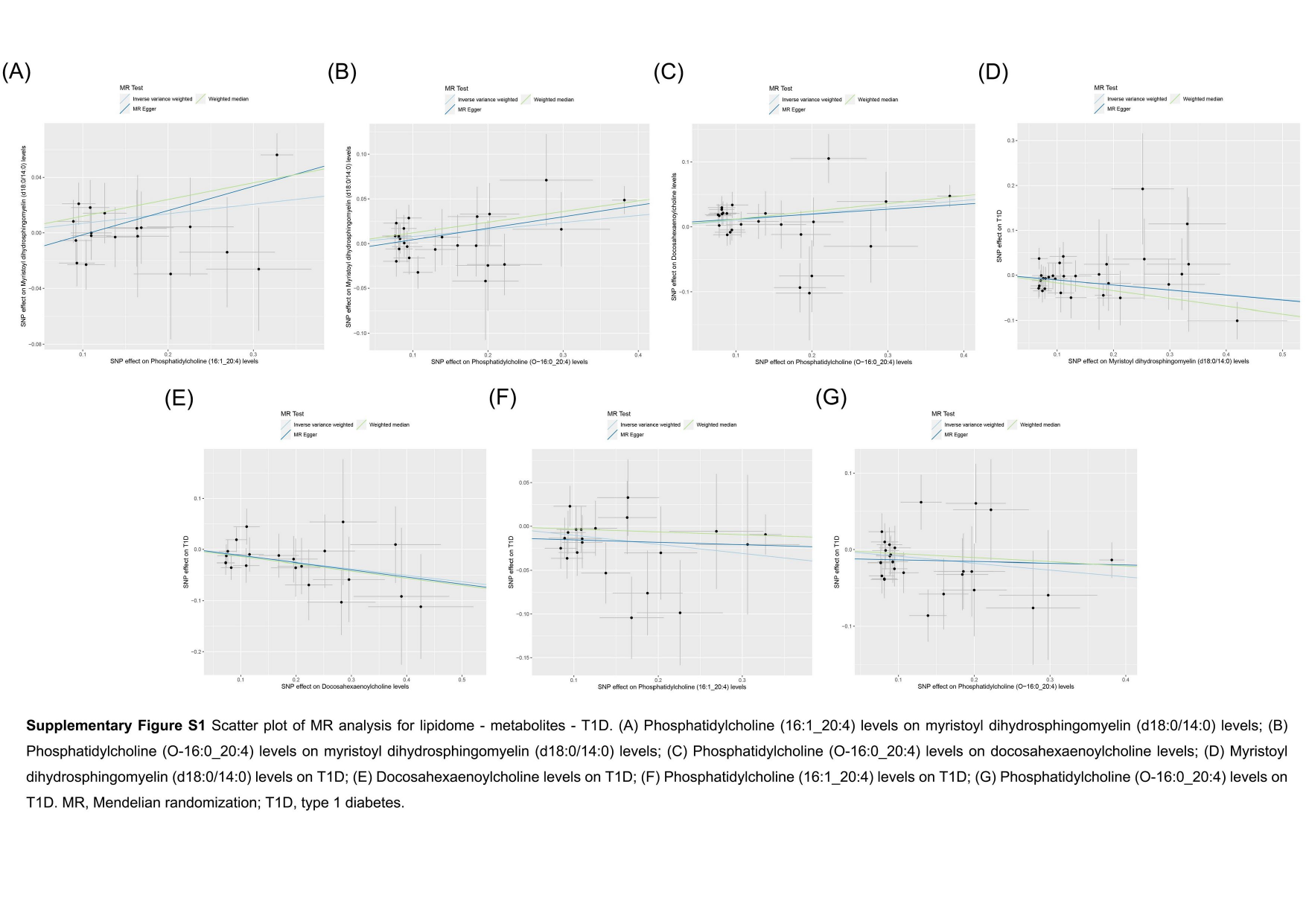

## Slide 2
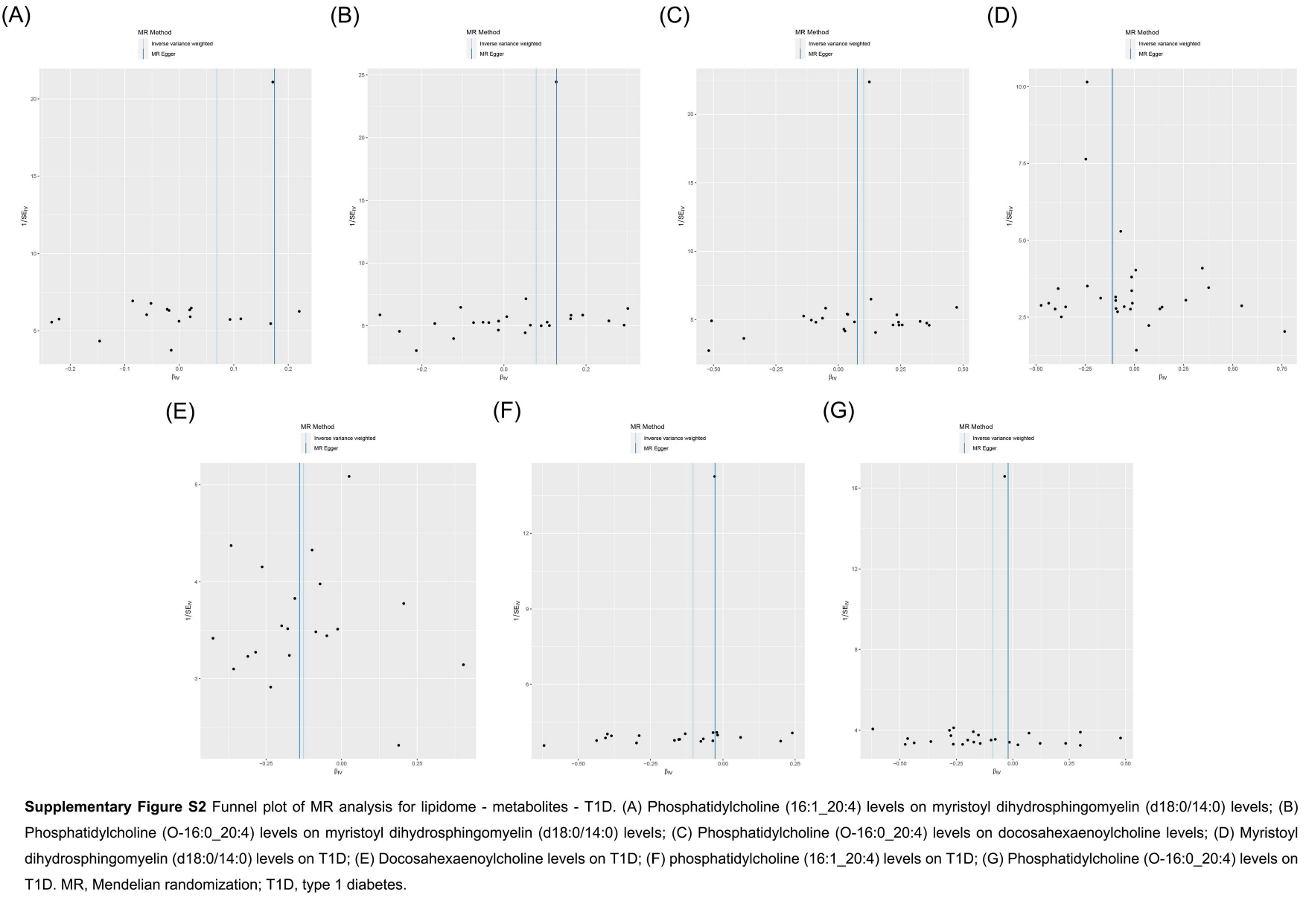

## Slide 3
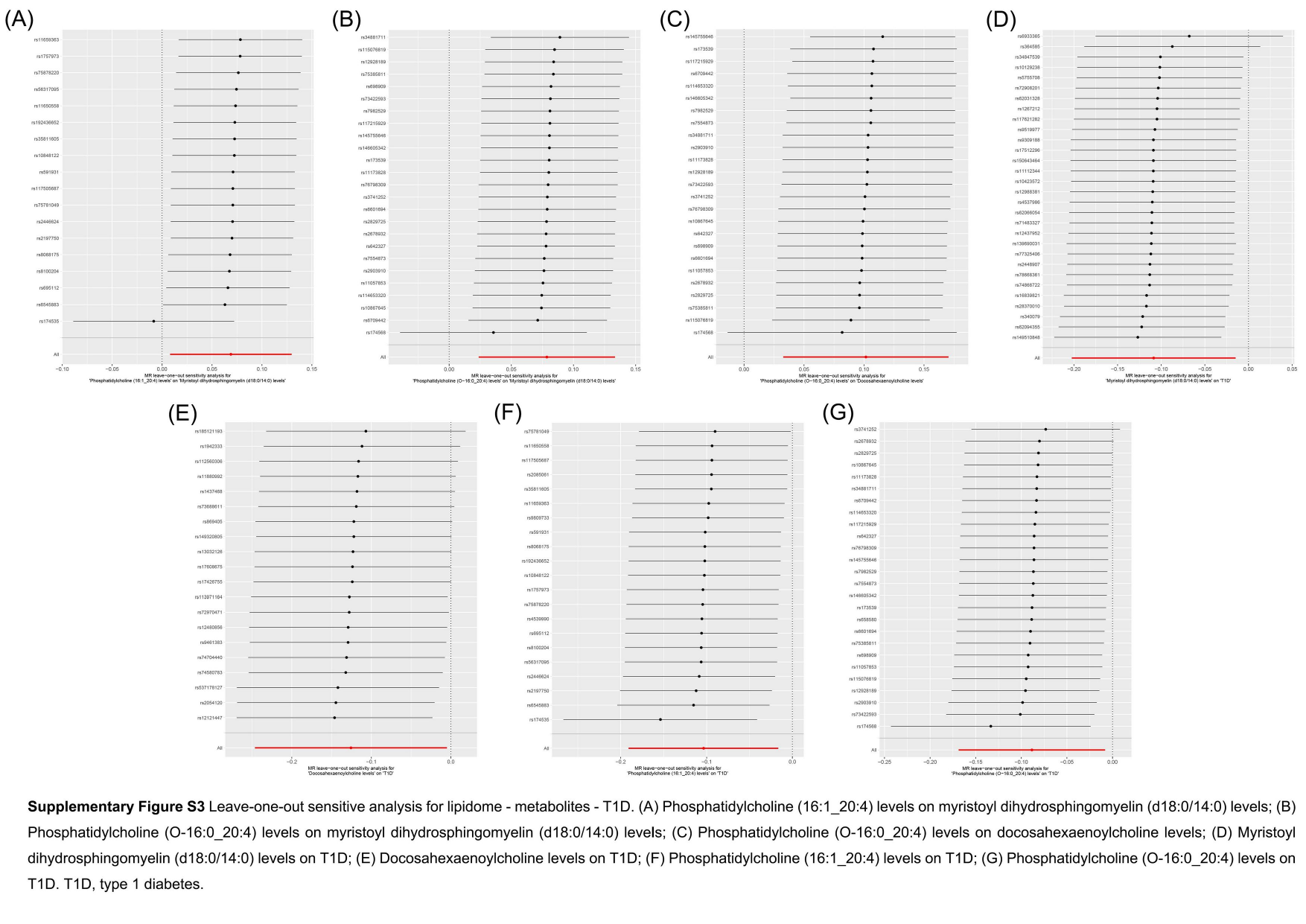

Supplement: Supplementary file 2 [file medi-104-e42755-s002.pptx]
